# Supplementary figures and images for: α1-Adrenergic receptor–PKC–Pyk2–Src signaling boosts L-type Ca2+ channel CaV1.2 activity and long-term potentiation in rodents
Source: eLife. 2023 Jun 20;12:e79648. doi: 10.7554/eLife.79648 (PMC10325713; doi:10.7554/eLife.79648)

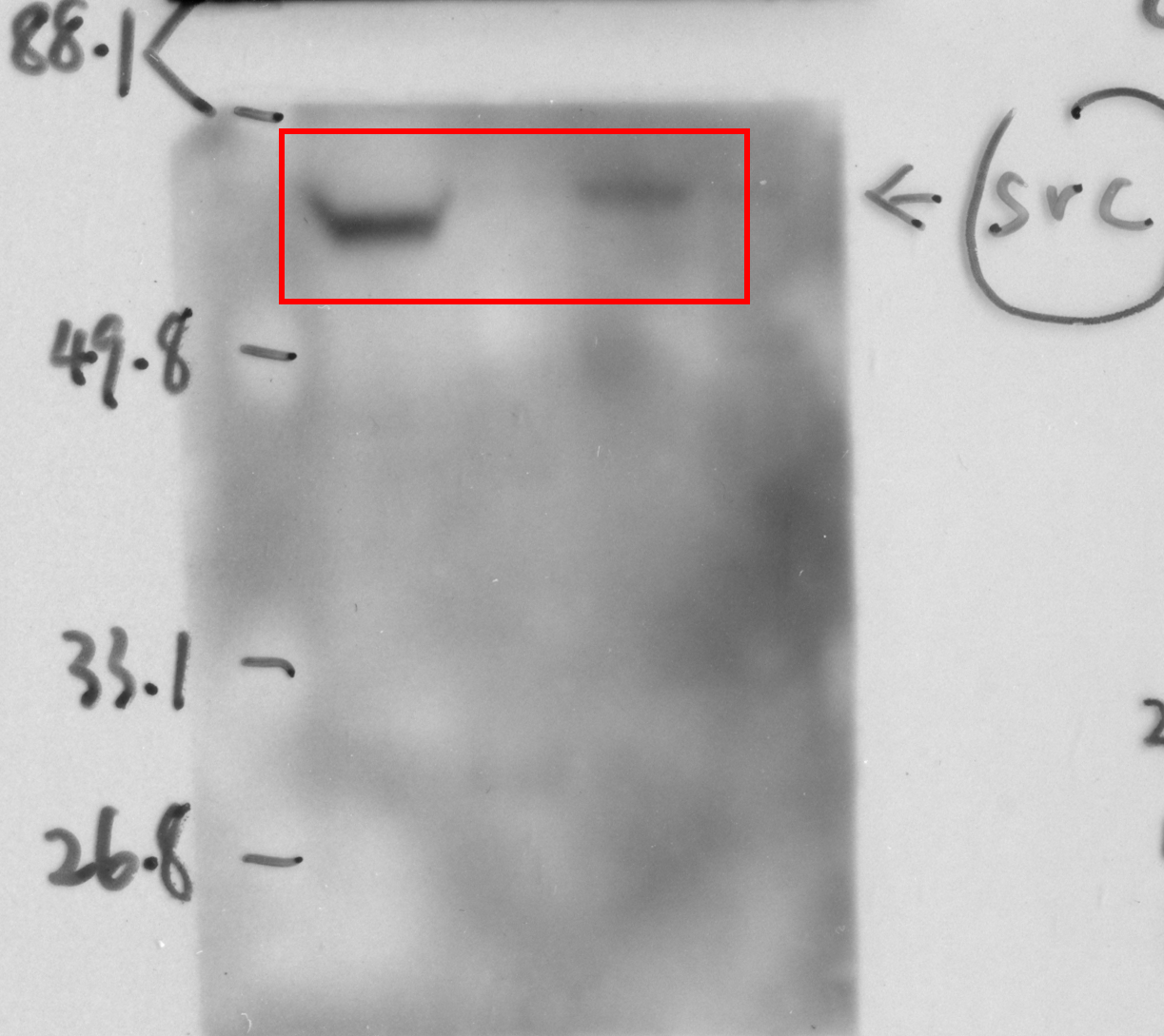

Supplement: Figure 7—source data 1. [file elife-79648-fig7-data1.zip › Figure 7-source data 1/Fig7A_LowerPanel_R.png]

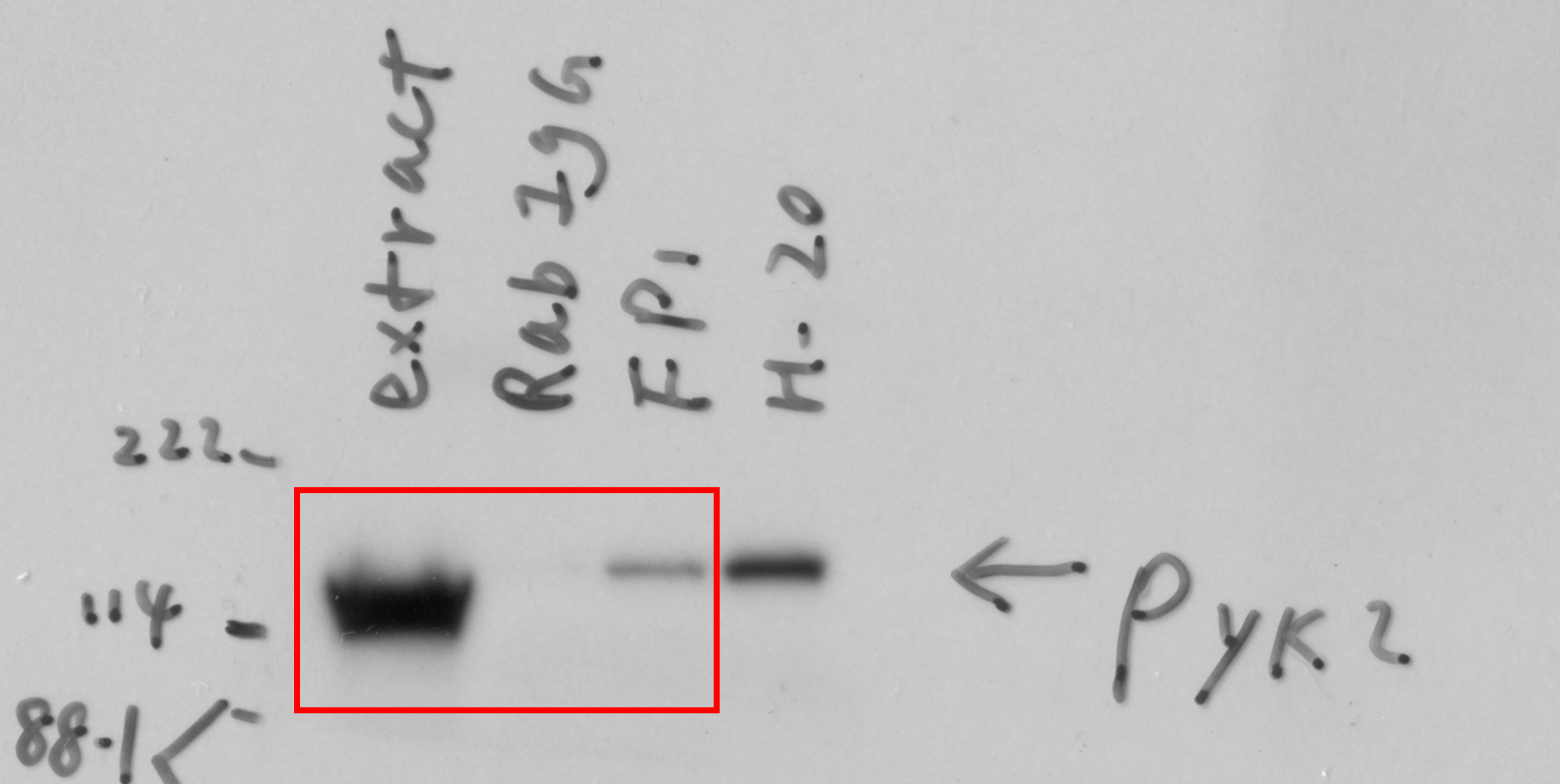

Supplement: Figure 7—source data 1. [file elife-79648-fig7-data1.zip › Figure 7-source data 1/Fig7A_UpperPanel_R.png]

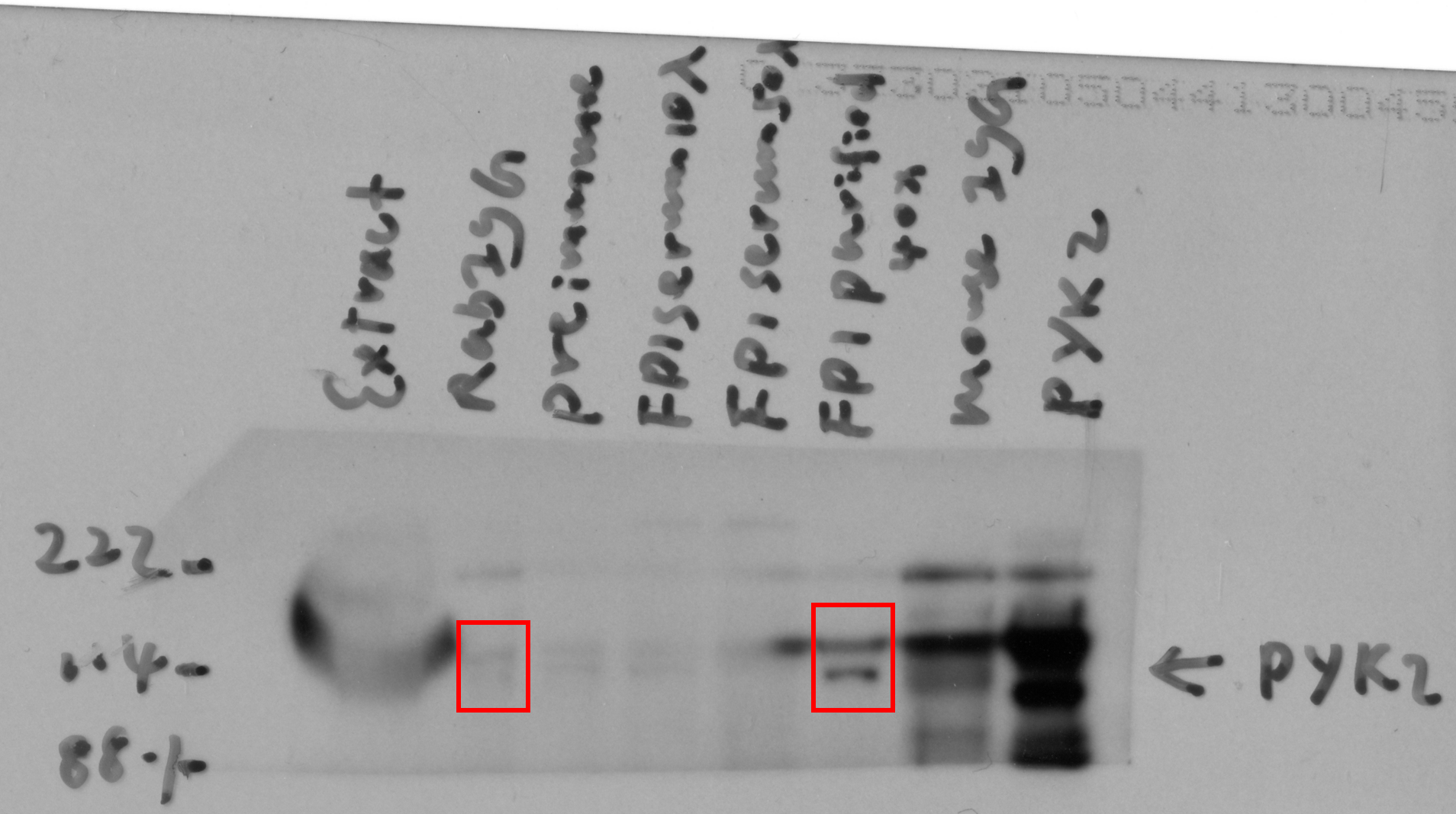

Supplement: Figure 7—source data 1. [file elife-79648-fig7-data1.zip › Figure 7-source data 1/Fig7B_Uncropped_Long_R.png]

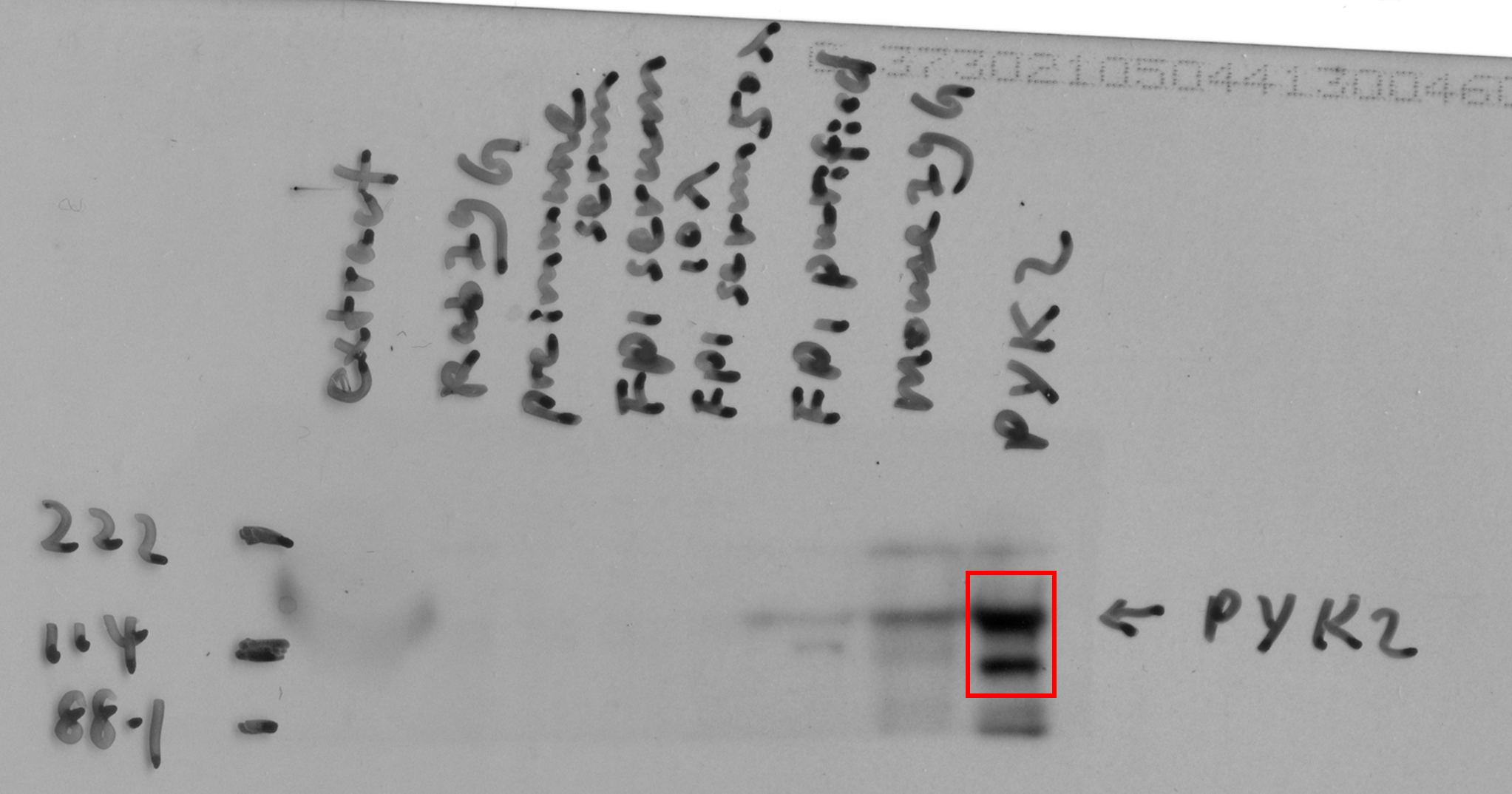

Supplement: Figure 7—source data 1. [file elife-79648-fig7-data1.zip › Figure 7-source data 1/Fig7B_Uncropped_Short_R.png]

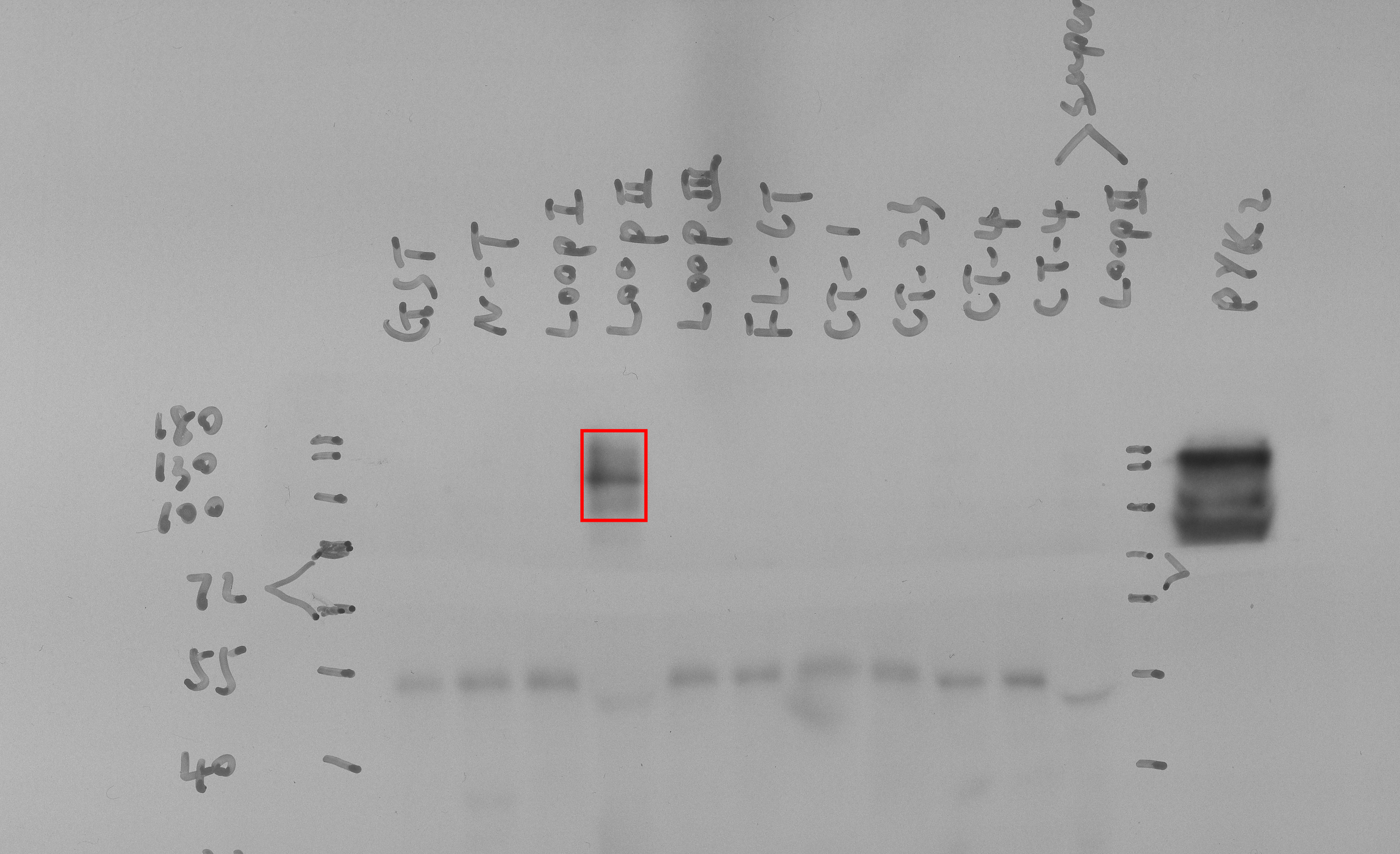

Supplement: Figure 7—source data 1. [file elife-79648-fig7-data1.zip › Figure 7-source data 1/Fig7D_R.png]

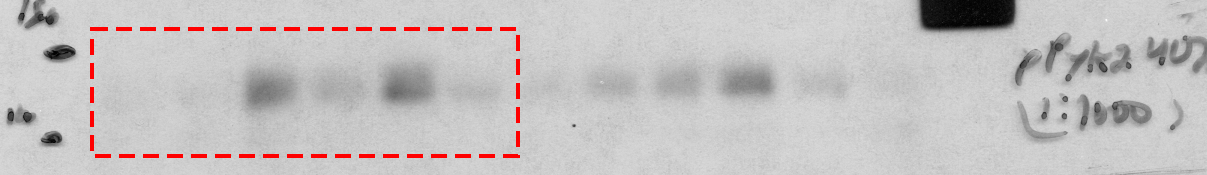

Supplement: Figure 8—source data 1. [file elife-79648-fig8-data1.zip › Figure 8-source data 1/Fig. 8B Boxed lanes in full blots/Fig. 8B Boxed lanes shown in full anti-pY402 Pyk2 in Lysate blot. tif.tif]

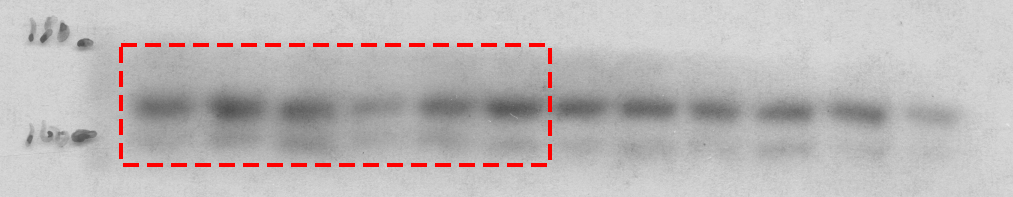

Supplement: Figure 8—source data 1. [file elife-79648-fig8-data1.zip › Figure 8-source data 1/Fig. 8B Boxed lanes in full blots/Fig. 8B boxed lanes shown in full blot anti-Pyk2 in Lysate.tif]

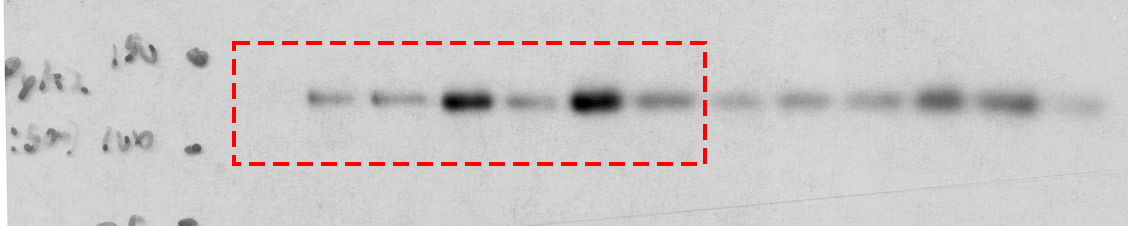

Supplement: Figure 8—source data 1. [file elife-79648-fig8-data1.zip › Figure 8-source data 1/Fig. 8B Boxed lanes in full blots/Fig. 8B boxed lanes shown of full anti-Pyk2 4G10 IP (top).tif]

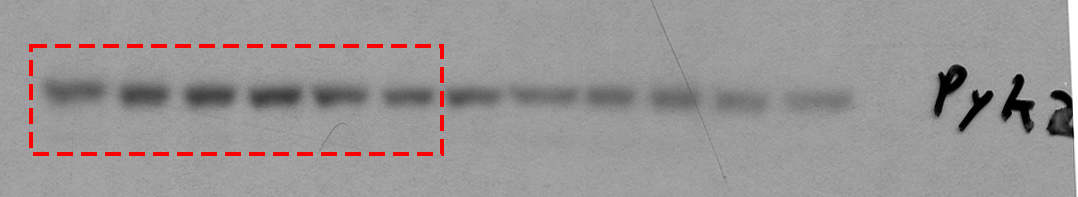

Supplement: Figure 8—source data 1. [file elife-79648-fig8-data1.zip › Figure 8-source data 1/Fig. 8E boxed Lanes in Full blot images/Fig. 8E anti-total Pyk2 blot 5-29-15 cresc 2 sec417.tif]

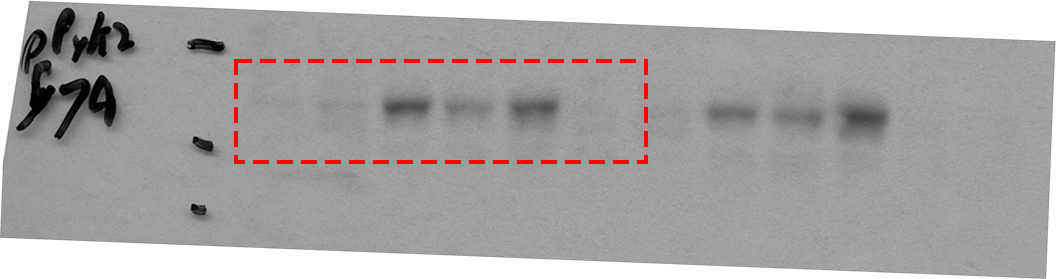

Supplement: Figure 8—source data 1. [file elife-79648-fig8-data1.zip › Figure 8-source data 1/Fig. 8E boxed Lanes in Full blot images/Fig. 8E boxed lanes in full blot anti-pY579 Pyk2 5 sec Lys.tif]

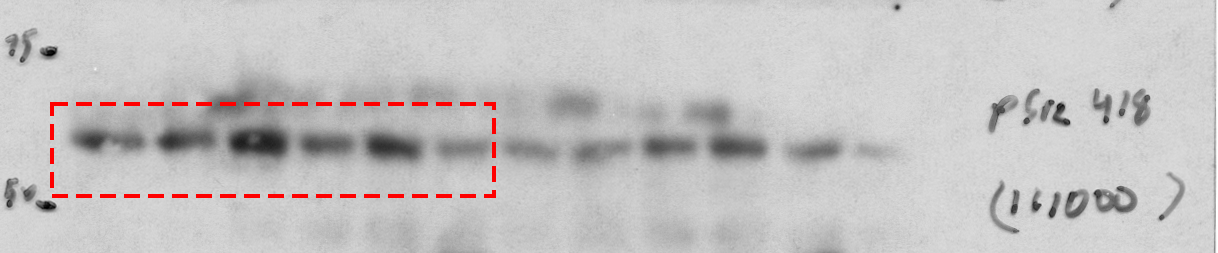

Supplement: Figure 8—source data 1. [file elife-79648-fig8-data1.zip › Figure 8-source data 1/Fig. 8G Full blots with Boxed Lanes shown in Fig/Fig. 8G Boxed Lanes of pTyr416Src 2-18-14 crescendo 20 sec blot.tif]

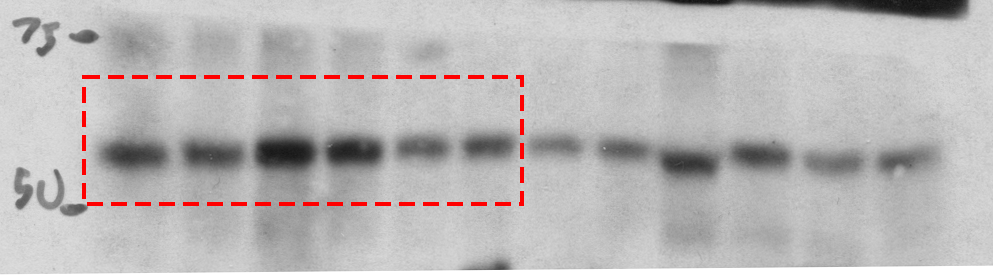

Supplement: Figure 8—source data 1. [file elife-79648-fig8-data1.zip › Figure 8-source data 1/Fig. 8G Full blots with Boxed Lanes shown in Fig/Fig. 8G Boxed lanes of Total Src 2-7-14 crescendo 10 sec126.tif]

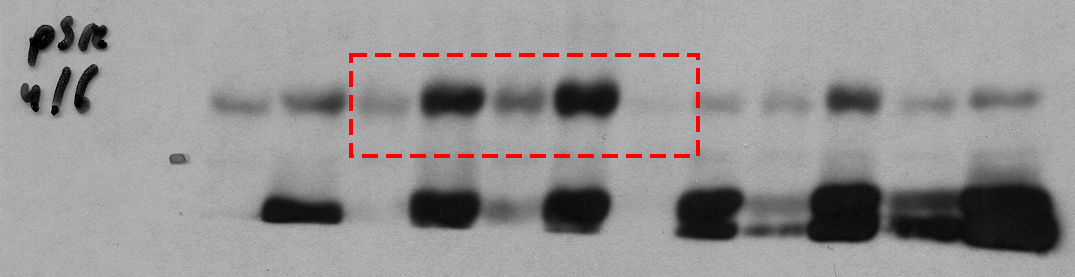

Supplement: Figure 8—source data 1. [file elife-79648-fig8-data1.zip › Figure 8-source data 1/Fig. 8I Boxed lanes in full blot/Boxed lanes in full blot 7-31-15 cresc 5 sec p416Src.tif]

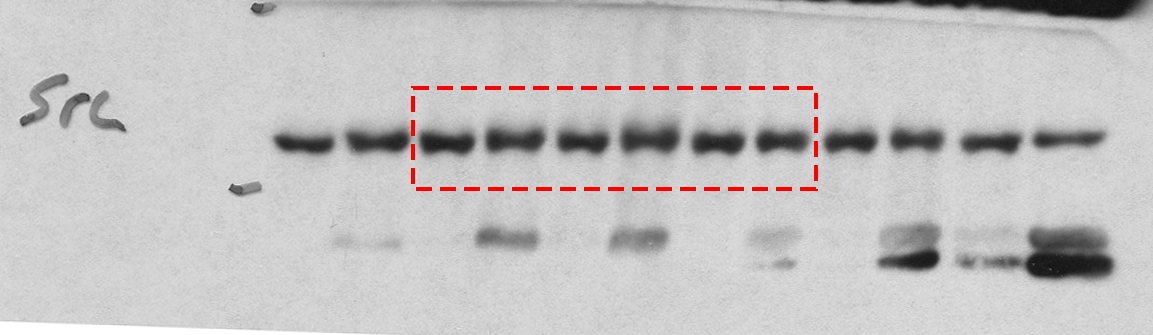

Supplement: Figure 8—source data 1. [file elife-79648-fig8-data1.zip › Figure 8-source data 1/Fig. 8I Boxed lanes in full blot/Fig. 8I Boxed lanes of8-1-15 cresc 10 min Total Src blot.tif]

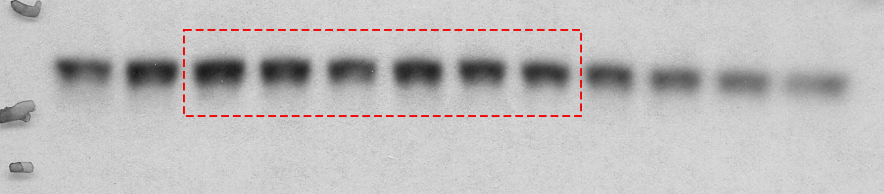

Supplement: Figure 8—source data 1. [file elife-79648-fig8-data1.zip › Figure 8-source data 1/Fig. 8K Boxed lanes of full images/Fig 8K Boxed lanes 8-1-15 cresc 5 sec Total Pyk2.tif]

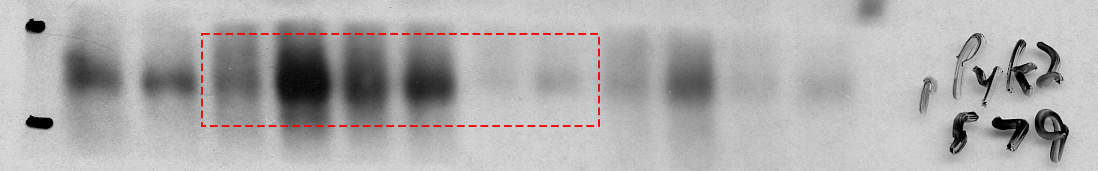

Supplement: Figure 8—source data 1. [file elife-79648-fig8-data1.zip › Figure 8-source data 1/Fig. 8K Boxed lanes of full images/Fig. 8K Boxed 7-23-15 cresc 7 sec p579Pyk2.tif]

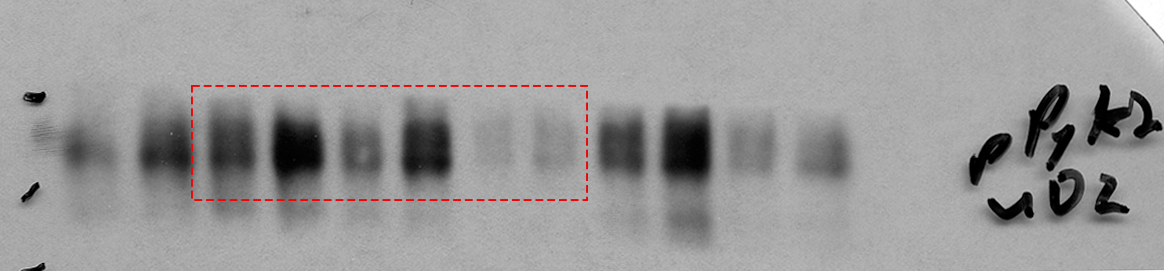

Supplement: Figure 8—source data 1. [file elife-79648-fig8-data1.zip › Figure 8-source data 1/Fig. 8K Boxed lanes of full images/Fig. 8K Boxed 7-24-15 cresc 5 sec p402Pyk2 .tif]

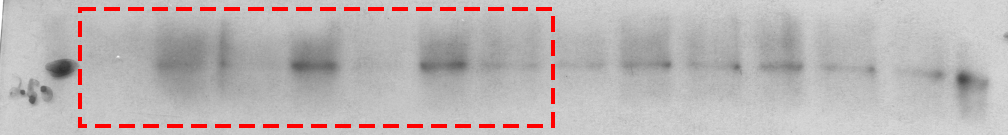

Supplement: Figure 9—source data 1. [file elife-79648-fig9-data1.zip › Figure 9-source data 1/Fig. 9 Boxed lanes used in Full Blots/Fig. 9B Boxed lanes in Fig. from 2-13-14 crescendo 10 sec 4G10-IP alpha1C.tif]

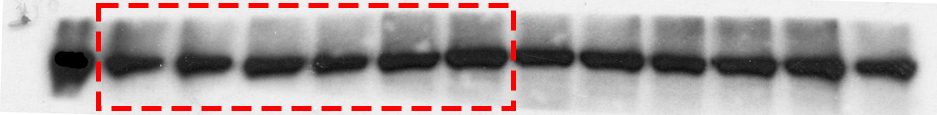

Supplement: Figure 9—source data 1. [file elife-79648-fig9-data1.zip › Figure 9-source data 1/Fig. 9 Boxed lanes used in Full Blots/Fig. 9B Boxed Lanes in full blot 2-13-14 crescendo 10 sec total alpha1C (FP1).tif]

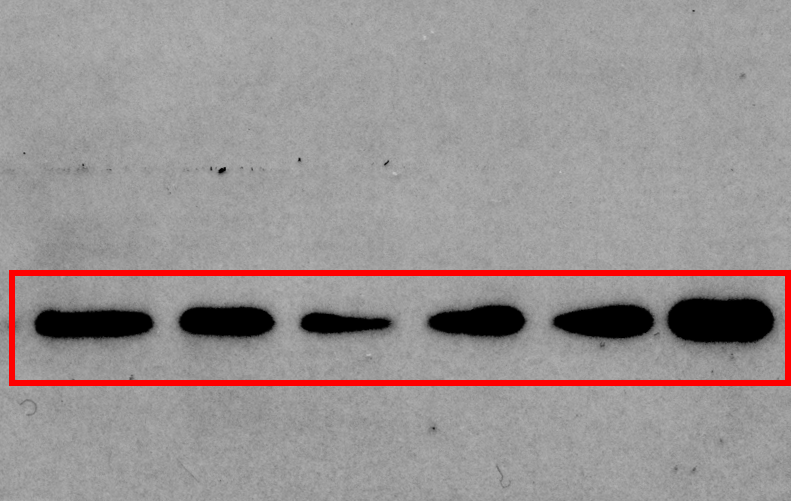

Supplement: Figure 9—source data 1. [file elife-79648-fig9-data1.zip › Figure 9-source data 1/Fig. 9 Boxed lanes used in Full Blots/Fig. 9D_LowerPanel_R.tif]

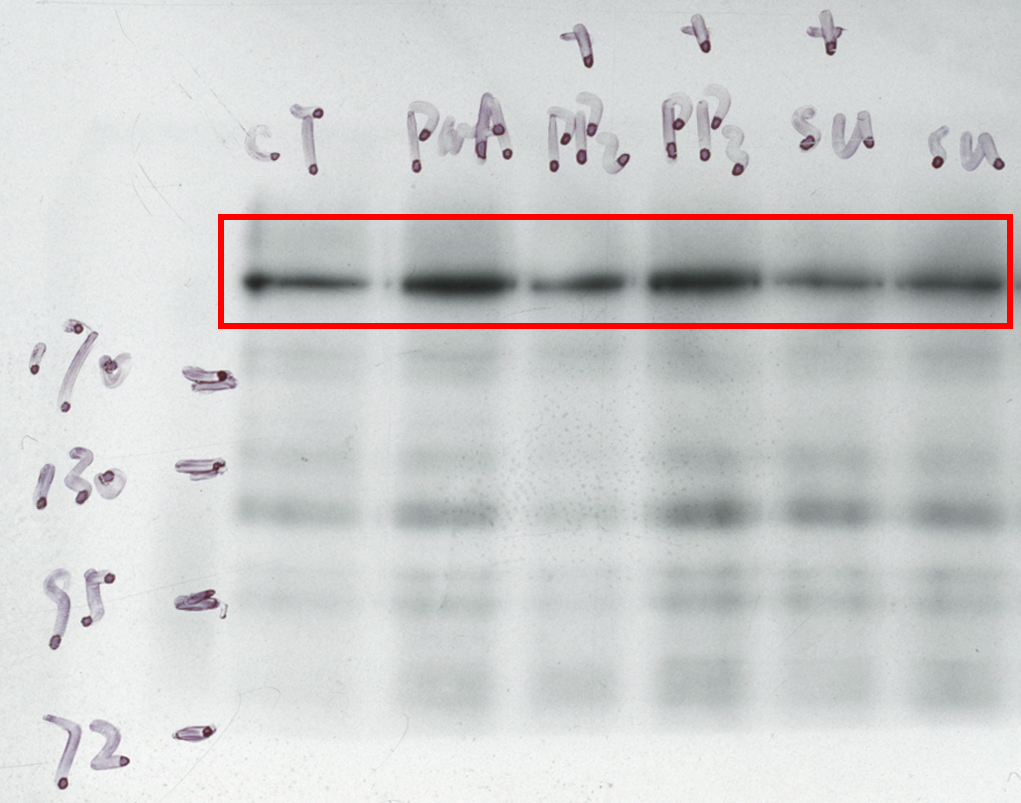

Supplement: Figure 9—source data 1. [file elife-79648-fig9-data1.zip › Figure 9-source data 1/Fig. 9 Boxed lanes used in Full Blots/Fig. 9D_UpperPanel_R.tif]

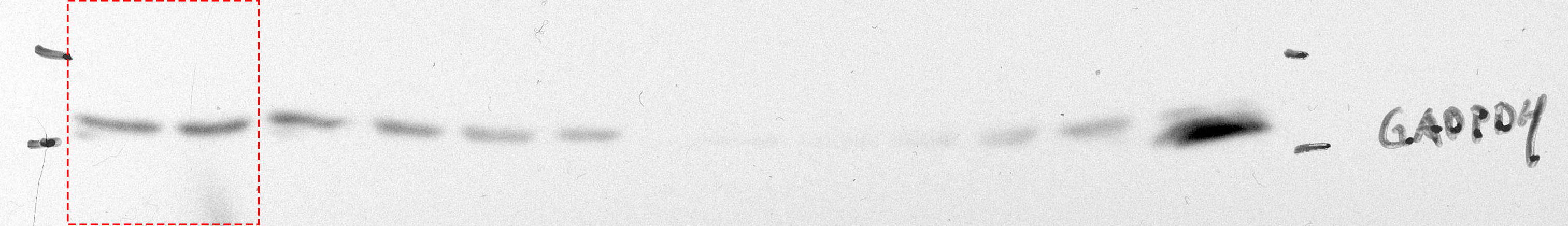

Supplement: Figure 10—source data 1. [file elife-79648-fig10-data1.zip › Figure 10-source data 1/Fig. 10A Boxed Lanes full scans/Boxed GAPDH lanes Fig. 10A .tif]

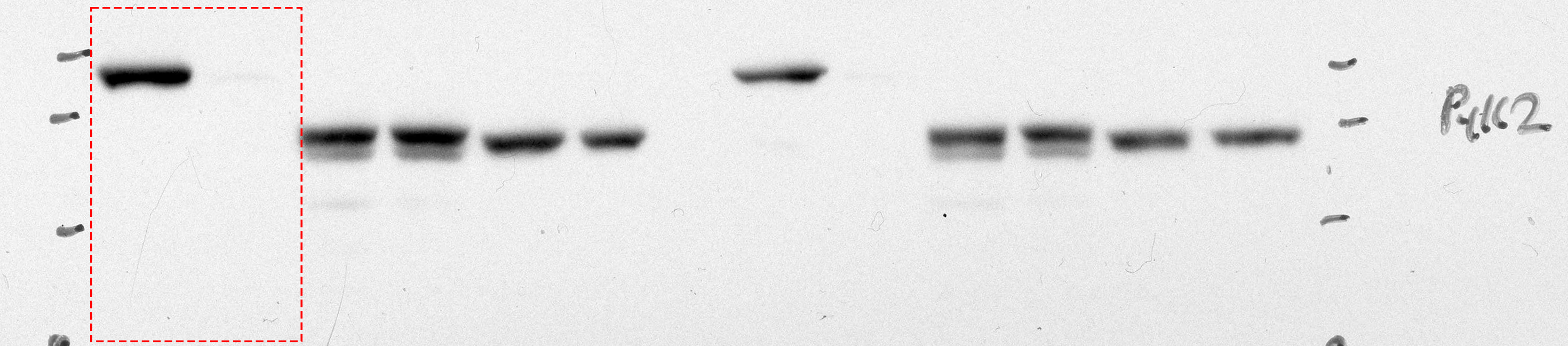

Supplement: Figure 10—source data 1. [file elife-79648-fig10-data1.zip › Figure 10-source data 1/Fig. 10A Boxed Lanes full scans/Boxed lanes Fig.10A anti-Pyk2 KD longer Exp .tif]

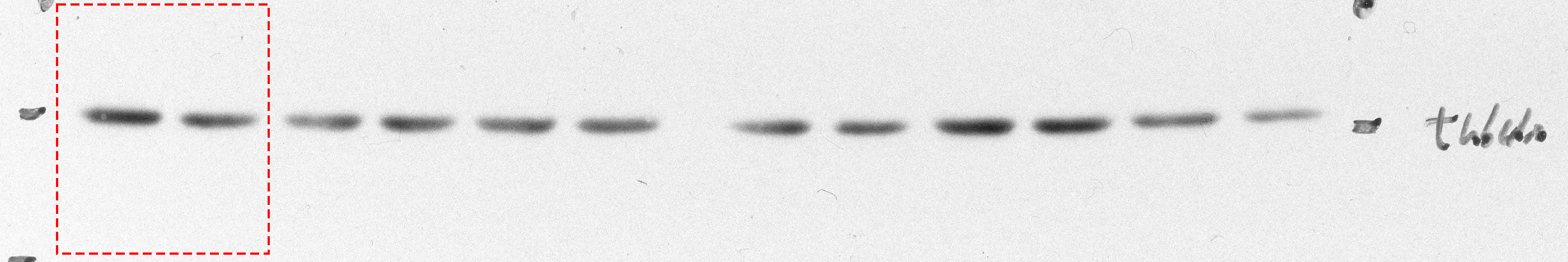

Supplement: Figure 10—source data 1. [file elife-79648-fig10-data1.zip › Figure 10-source data 1/Fig. 10A Boxed Lanes full scans/Fig. 10A Boxed lanes all tubulin.tif]

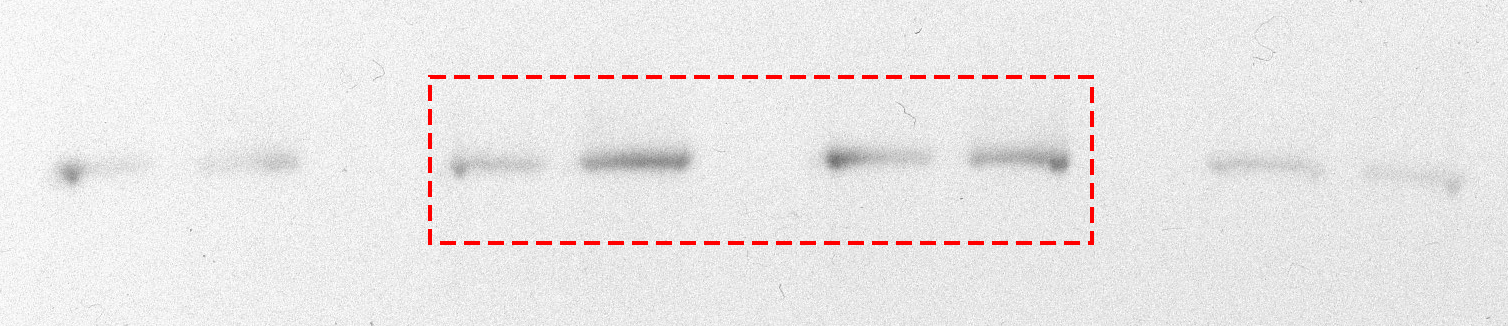

Supplement: Figure 10—source data 1. [file elife-79648-fig10-data1.zip › Figure 10-source data 1/Fig. 10B Boxed Lanes Full blot Scans/Boxed lanes Fig. 10B 20111104 PMA shPyk2 4G10IP FP1IB.tif]

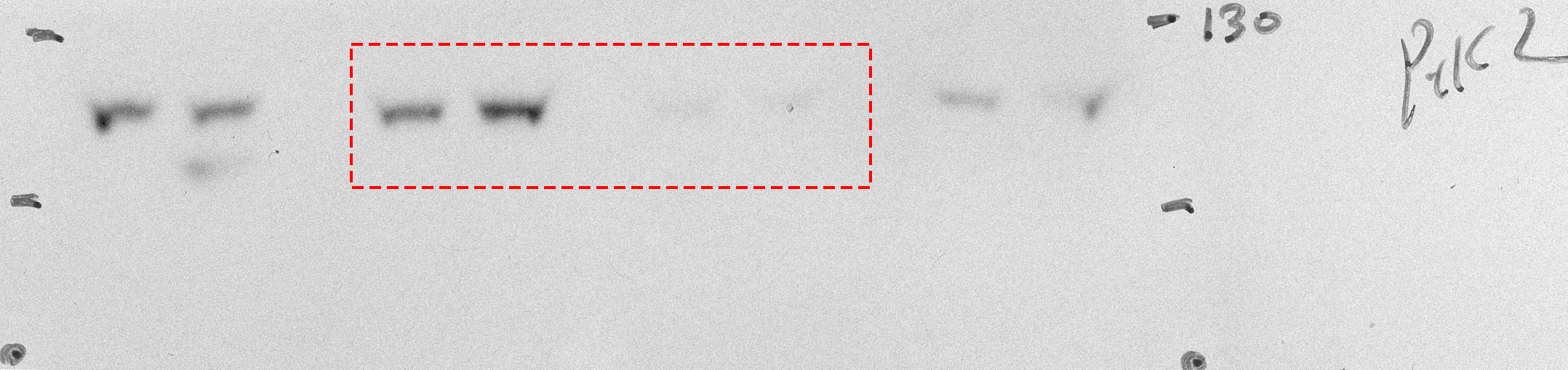

Supplement: Figure 10—source data 1. [file elife-79648-fig10-data1.zip › Figure 10-source data 1/Fig. 10B Boxed Lanes Full blot Scans/Boxed PMA shPyk2 lys Pyk2 IB2.tif]

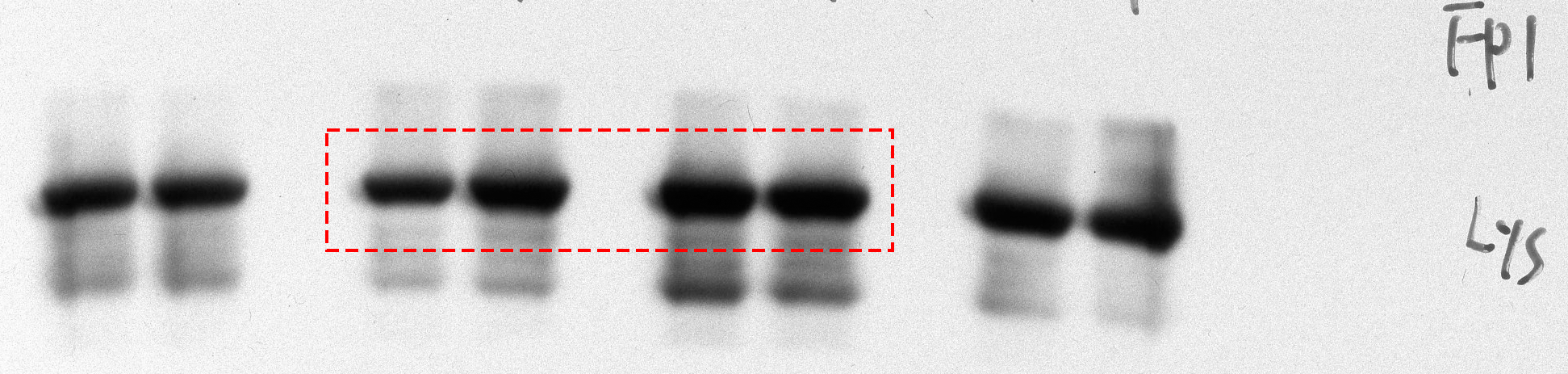

Supplement: Figure 10—source data 1. [file elife-79648-fig10-data1.zip › Figure 10-source data 1/Fig. 10B Boxed Lanes Full blot Scans/Fig. 10B Boxed lanes 20111104 PMA shPyk2 FP1IB Lys.tif]

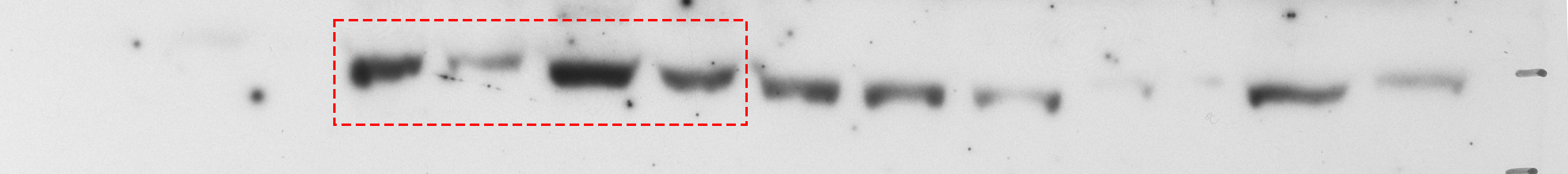

Supplement: Figure 10—source data 1. [file elife-79648-fig10-data1.zip › Figure 10-source data 1/Fig. 10C Boxed lanes Film scans/Boxed lanes Crop to 052212 BK shKD Pyk2 Lys022.tif]

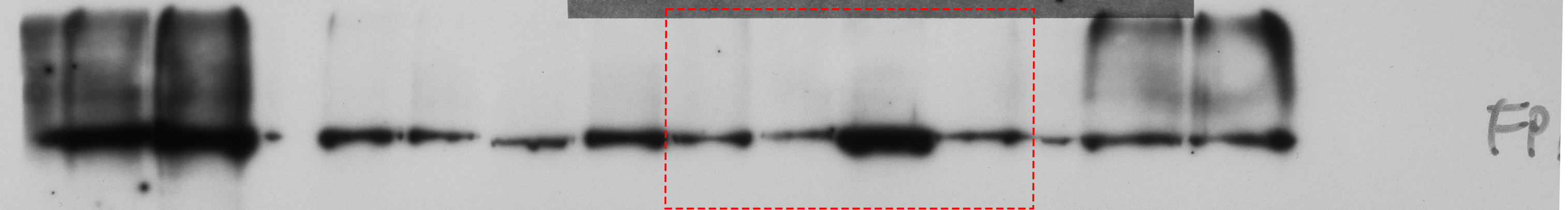

Supplement: Figure 10—source data 1. [file elife-79648-fig10-data1.zip › Figure 10-source data 1/Fig. 10C Boxed lanes Film scans/Boxed lanes Fig. 10C +- BK shKD 4G10 IP FP1 anti-alpa1C.tif]

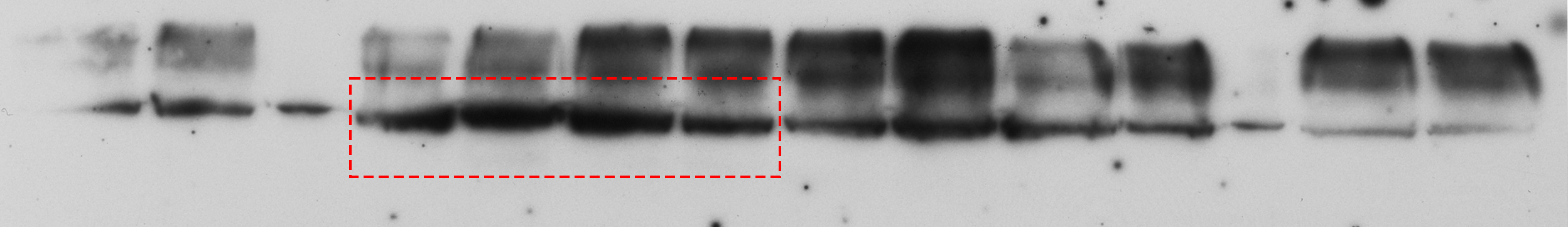

Supplement: Figure 10—source data 1. [file elife-79648-fig10-data1.zip › Figure 10-source data 1/Fig. 10C Boxed lanes Film scans/Boxed Short Exp +- BK shKD FP1 anti-a1C Lys tif.tif]

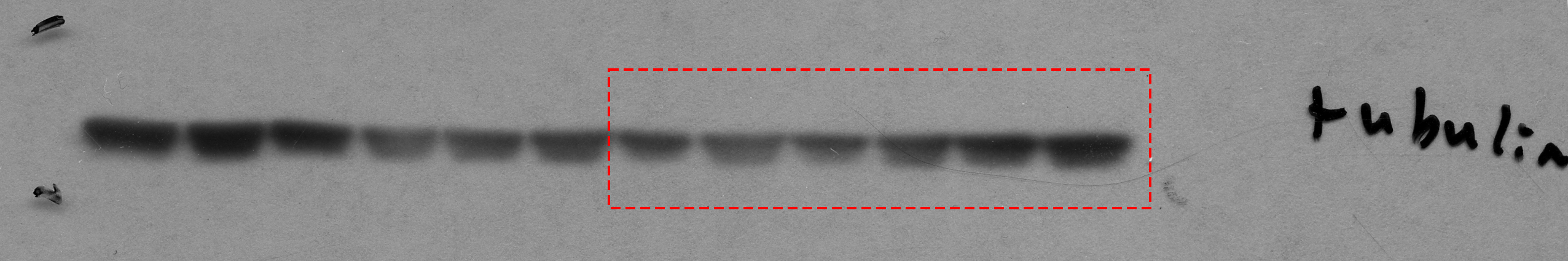

Supplement: Figure 10—source data 1. [file elife-79648-fig10-data1.zip › Figure 10-source data 1/Fig. 10E Boxed lanes full Scan images/Fig. 10 E Boxed lanes 6 Sec anti-alpha tubulin.tif]

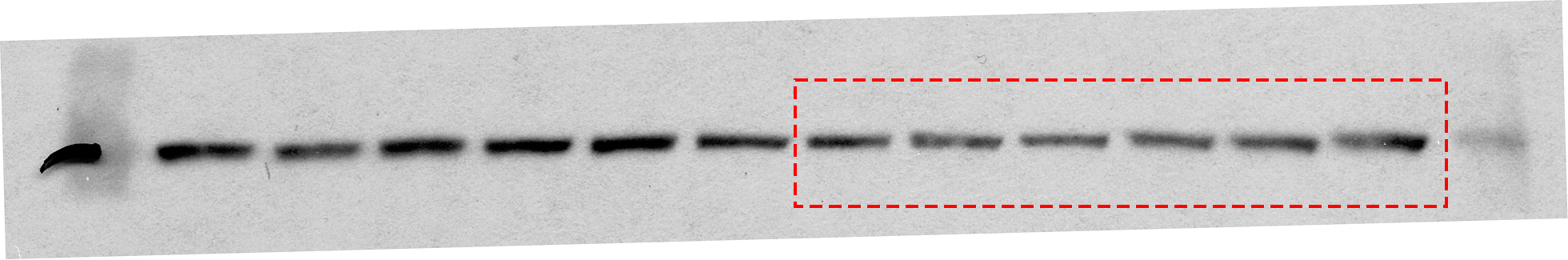

Supplement: Figure 10—source data 1. [file elife-79648-fig10-data1.zip › Figure 10-source data 1/Fig. 10E Boxed lanes full Scan images/Fig. 10E Boxed lanes full scan 4sec anti-a1C (FP1).tif]

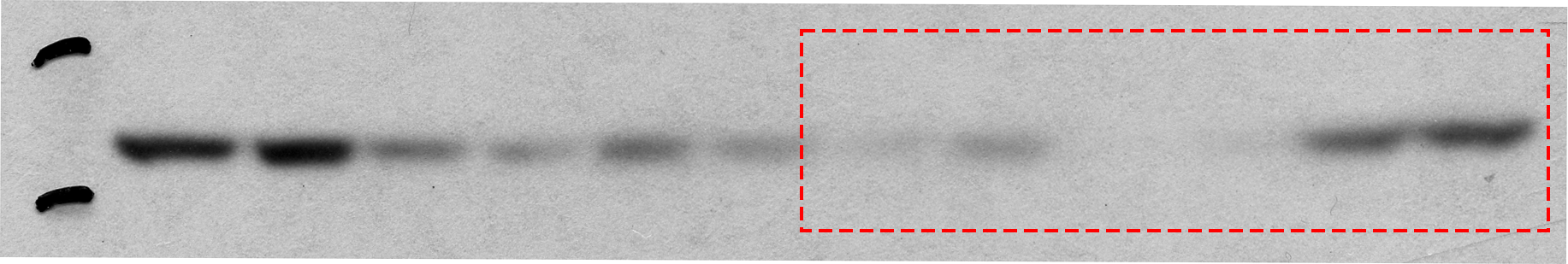

Supplement: Figure 10—source data 1. [file elife-79648-fig10-data1.zip › Figure 10-source data 1/Fig. 10E Boxed lanes full Scan images/Fig. 10E Boxed Lanes PyK2 only 4-22-16 class 20 sec026 copy.tif]

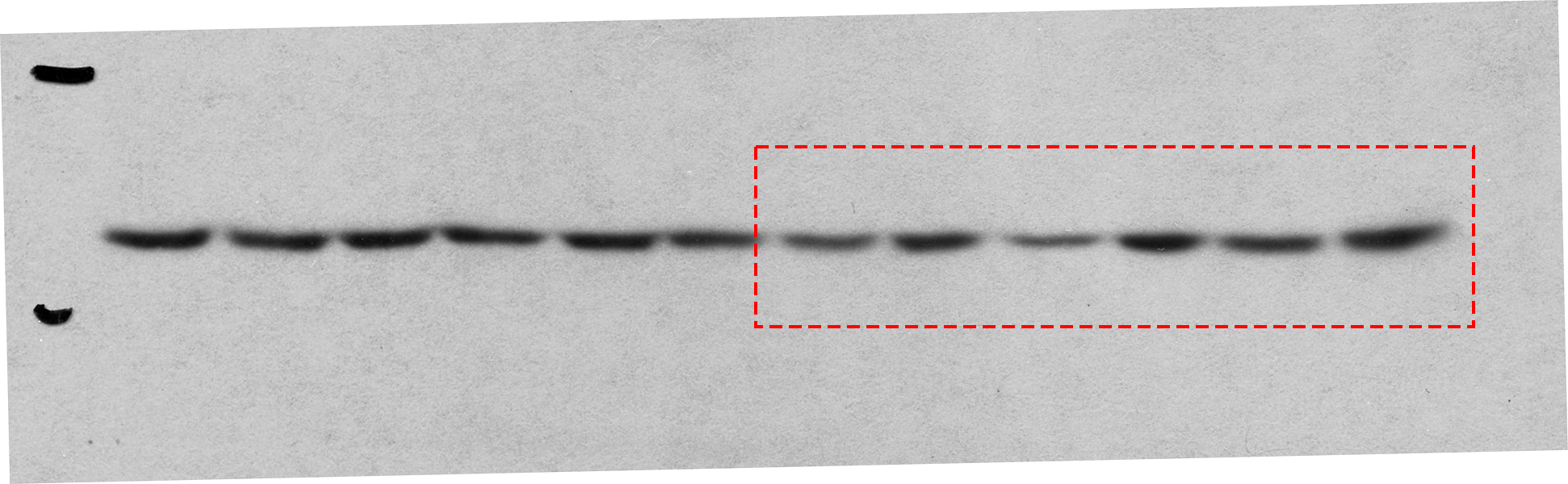

Supplement: Figure 10—source data 1. [file elife-79648-fig10-data1.zip › Figure 10-source data 1/Fig. 10E Boxed lanes full Scan images/Fig. 10E Src Boxed lanes Scan of anti-Src.tif]

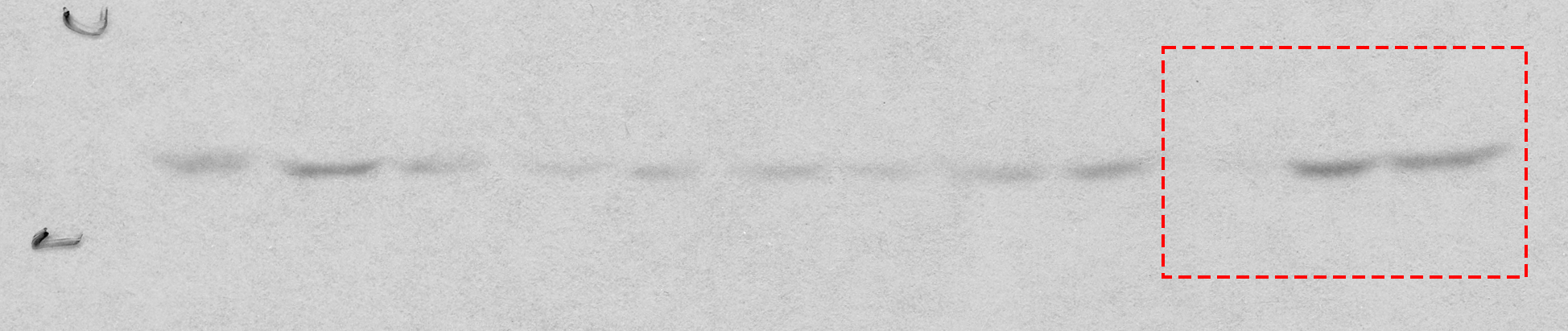

Supplement: Figure 10—source data 1. [file elife-79648-fig10-data1.zip › Figure 10-source data 1/Fig. 10F Boxed lanes of full blot scans/Fig. 10F Left Boxed Lanes 4-29-16 anti-Src IB lys 20 sec.tif]

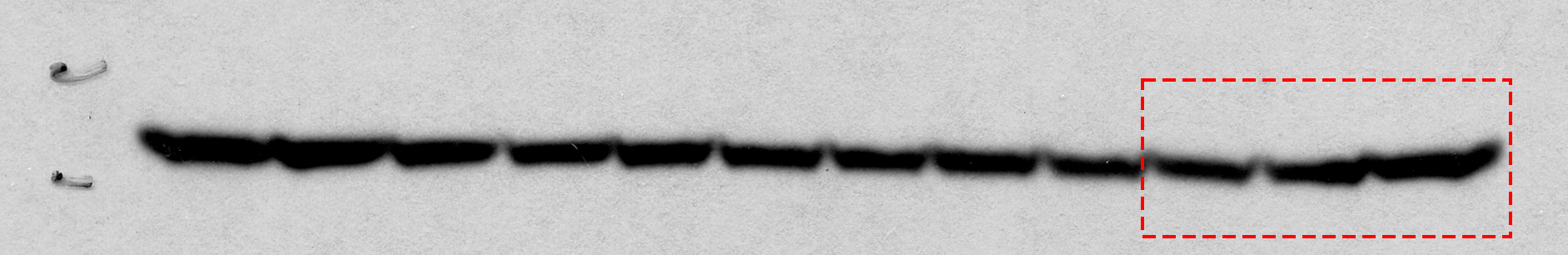

Supplement: Figure 10—source data 1. [file elife-79648-fig10-data1.zip › Figure 10-source data 1/Fig. 10F Boxed lanes of full blot scans/Fig. 10F Left Boxed lanes anti-PyK2 lys 20 sec.tif]

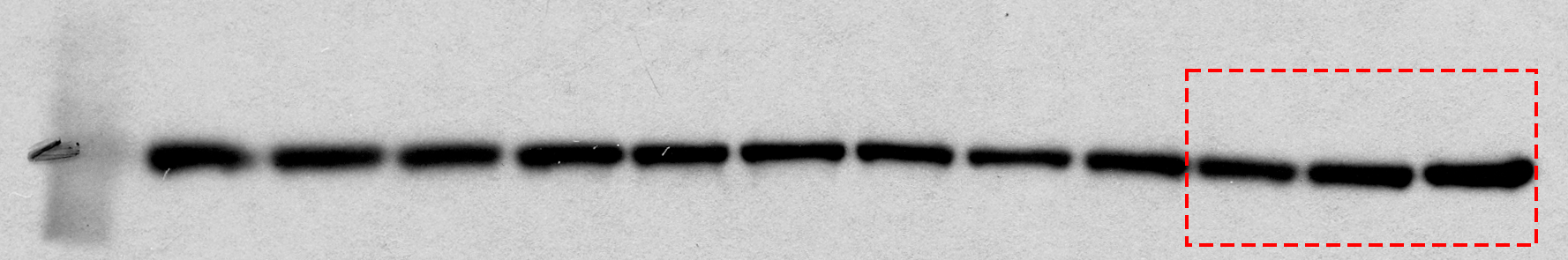

Supplement: Figure 10—source data 1. [file elife-79648-fig10-data1.zip › Figure 10-source data 1/Fig. 10F Boxed lanes of full blot scans/Fig. 10F Left Boxed Lanes FP1 anti-a1C 4-29-16 20 sec.tif]

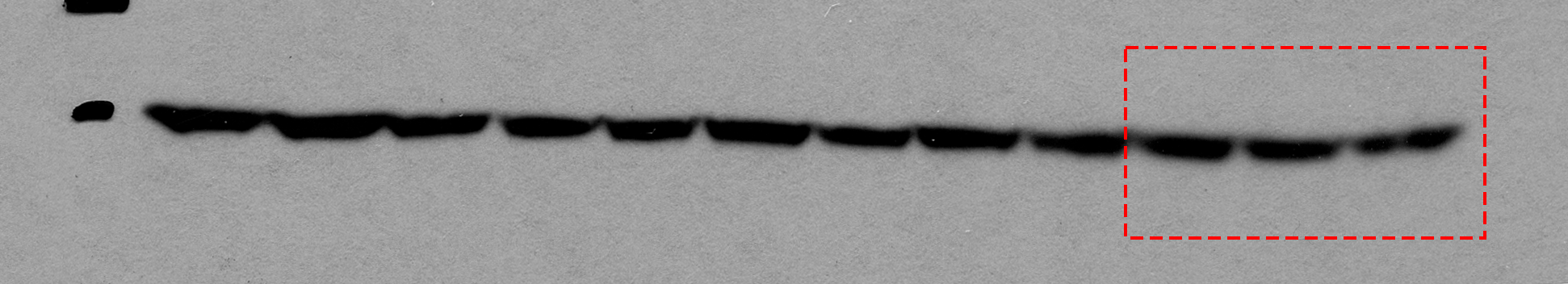

Supplement: Figure 10—source data 1. [file elife-79648-fig10-data1.zip › Figure 10-source data 1/Fig. 10F Boxed lanes of full blot scans/Fig. 10F Left Boxed lanes Vinculin 5-3-16 cresc 20 sec.tif]

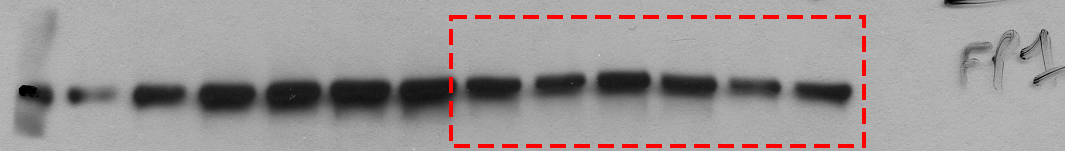

Supplement: Figure 10—source data 1. [file elife-79648-fig10-data1.zip › Figure 10-source data 1/Fig. 10F Boxed lanes of full blot scans/Fig. 10F Right Boxed lanes Full FP1 anti a1C blot For Src KD-B 9-27-16 cresc 20 sec401.tif]

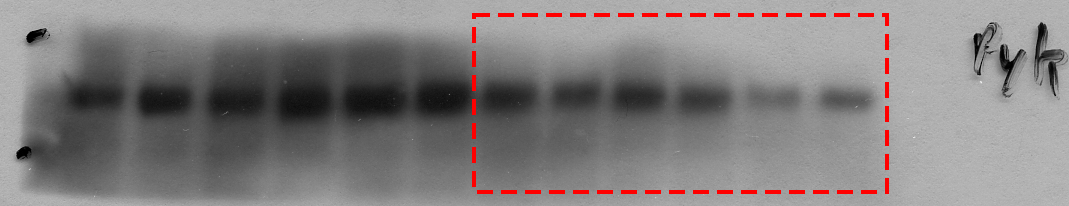

Supplement: Figure 10—source data 1. [file elife-79648-fig10-data1.zip › Figure 10-source data 1/Fig. 10F Boxed lanes of full blot scans/Fig. 10F right Boxed lanes Full tot Lys Pyk2 blot for Src KD-B 9-27-16 cresc 20 sec401.tif]

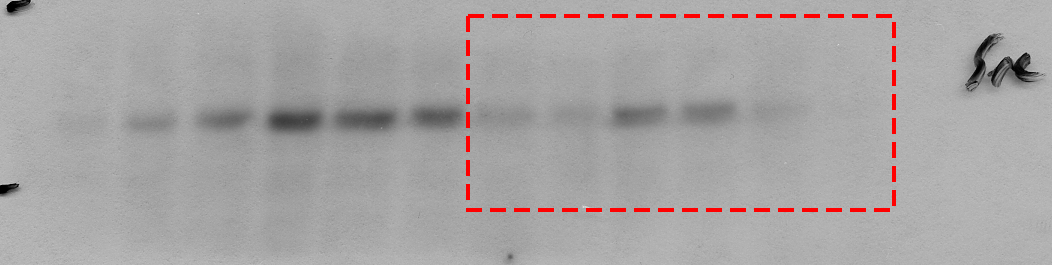

Supplement: Figure 10—source data 1. [file elife-79648-fig10-data1.zip › Figure 10-source data 1/Fig. 10F Boxed lanes of full blot scans/Fig. 10F Right Boxed lanes Src blot KD-B 9-27-16 cresc 20 sec401.tif]

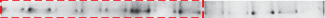

Supplement: Figure 10—source data 1. [file elife-79648-fig10-data1.zip › Figure 10-source data 1/Fig. 10G full blot images with boxed lanes /Fig 10 G Boxed lanes Full image all lanes 4G10 IP FP1 blot (top).tif]

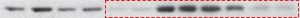

Supplement: Figure 10—source data 1. [file elife-79648-fig10-data1.zip › Figure 10-source data 1/Fig. 10G full blot images with boxed lanes /Fig 10 G Boxed Lanes Full image all lanes Py2 blot Lysate (mid).tif]

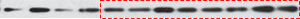

Supplement: Figure 10—source data 1. [file elife-79648-fig10-data1.zip › Figure 10-source data 1/Fig. 10G full blot images with boxed lanes /Fig 10G Boxed Lanes Full image all lanes FP1 blot Lysate (top).tif]

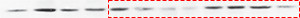

Supplement: Figure 10—source data 1. [file elife-79648-fig10-data1.zip › Figure 10-source data 1/Fig. 10G full blot images with boxed lanes /Fig 10G Boxed lanes Full image all lanes Src blot Lysate (botm).tif]

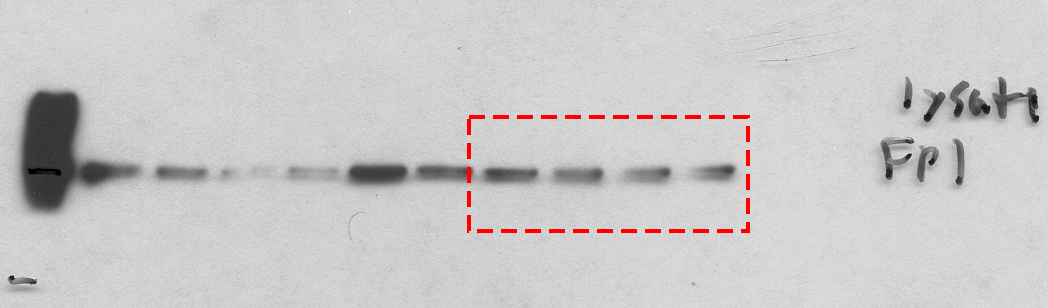

Supplement: Figure 10—source data 1. [file elife-79648-fig10-data1.zip › Figure 10-source data 1/Fig. 10H Boxed lanes full blot scans/Fig. 10H Boxed Lanes FP1 Tot Lys 5-12-16 cresc 7sec.tif]

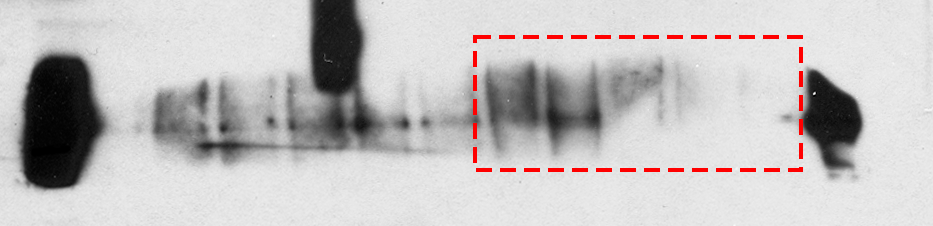

Supplement: Figure 10—source data 1. [file elife-79648-fig10-data1.zip › Figure 10-source data 1/Fig. 10H Boxed lanes full blot scans/Fig. 10H Boxed lanes full 4G10 IP FP1 5-11-16 femto 1 min copy.tif]

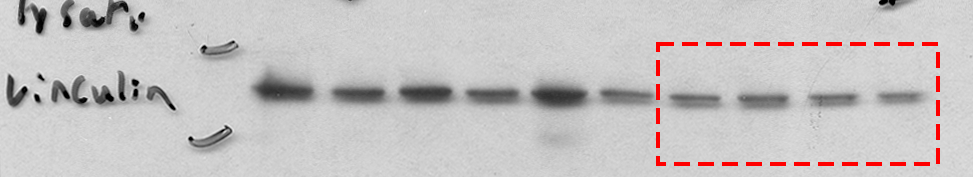

Supplement: Figure 10—source data 1. [file elife-79648-fig10-data1.zip › Figure 10-source data 1/Fig. 10H Boxed lanes full blot scans/Fig. 10H Boxed Lanes full scan Vinculin only in Tot Lys 5-13-16 cresc 1min.tif]

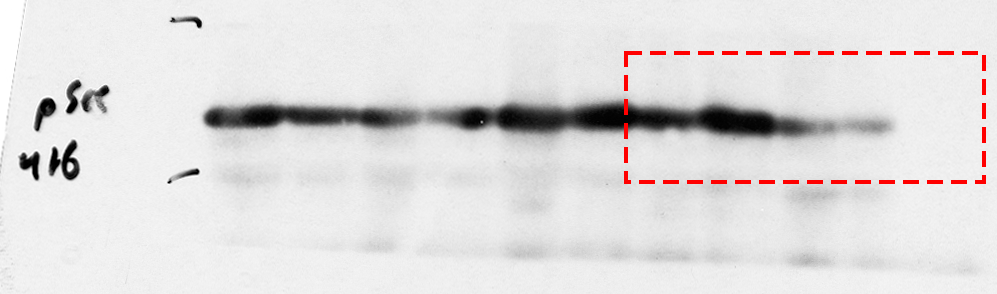

Supplement: Figure 10—source data 1. [file elife-79648-fig10-data1.zip › Figure 10-source data 1/Fig. 10H Boxed lanes full blot scans/Fig. 10H Boxed Lanes Src416 4G10 IP5-11-16 classico 3sec.tif]
