# Supplementary material for: α1-Adrenergic receptor–PKC–Pyk2–Src signaling boosts L-type Ca2+ channel CaV1.2 activity and long-term potentiation in rodents
Source: eLife. 2023 Jun 20;12:e79648. doi: 10.7554/eLife.79648 (PMC10325713; doi:10.7554/eLife.79648)
Supplement: Supplementary file 1. [file elife-79648-supp1.docx]

Supplementary File 1.

| **Designation** | **Residues*** | **Species** |
| --- | --- | --- |
| N-term | 2-124 | rat |
| Loop I-II | 409-526 | rat |
| Loop II-III | 754-901 | rat |
| Loop III-IV | 1165-1219 | rat |
| C-term | 1584-2140 | rat |
| CT-1 | 1507-1733 | rabbit |
| CT-23 | 1622-1905 | rabbit |
| CT-4 | 1909-2171 | rabbit |

* residue number corresponds to the initial α_1_1.2 sequence, which originated from rabbit heart (Mikami et al., 1989) (Gene Bank Accession number: CAA33546, NM_001136522); rat numbers correspond to α_1_1.2 as originally cloned from brain (Snutch et al., 1990) (Gene Bank Accession number: NM_012517).

**Supplementary File 1. Amino acid residues of fragments of intracellular loops of Cav1.2 α_1_-subunit used in GST pull-down studies**
