# Supplementary material for: α1-Adrenergic receptor–PKC–Pyk2–Src signaling boosts L-type Ca2+ channel CaV1.2 activity and long-term potentiation in rodents
Source: eLife. 2023 Jun 20;12:e79648. doi: 10.7554/eLife.79648 (PMC10325713; doi:10.7554/eLife.79648)
Supplement: Supplementary file 2. [file elife-79648-supp2.docx]

Supplementary File 2.

| **Antibody** | **Used at dilution** |
| --- | --- |
| Anti-Cav1.2 α1-subunit (raised in the lab) | 1:10000 |
| Anti-Pyk2 (Millipore Cat#05-488; RRID:AB_2174219) | 1:5000 |
| Anti-Pyk2 phospho-Y402 (Millipore Cat# 07-892; RRID:AB_568885) | 1:20000 |
| Anti-Pyk2 phospho-Y579 (Life Technologies Cat#44-632G) | 1:10000 |
| Anti-Src (gift from J.S. Brugge) | 1:500 |
| Anti-Src phospho-Y416 (Millipore Cat# 05-857; RRID:AB_441924) | 1:10000 |
| Anti-α-tubulin | 1:40000 |
| Anti-GAPDH | 1:10000 |

**Supplementary File 2. Antibody dilutions used for immunoblotting.**
