# Supplementary material for: α1-Adrenergic receptor–PKC–Pyk2–Src signaling boosts L-type Ca2+ channel CaV1.2 activity and long-term potentiation in rodents
Source: eLife. 2023 Jun 20;12:e79648. doi: 10.7554/eLife.79648 (PMC10325713; doi:10.7554/eLife.79648)
Supplement: Supplementary file 3. [file elife-79648-supp3.docx]

**Supplementary File 3.**

| **Name of shRNA** | **Sequence** |
| --- | --- |
| Sh1 | GAT GTA GTT CTT AAC CGC A |
| Pyk2-ShB | ACC AGG AGA CCT ATC GCT GTG AAC TCA TT |
| Pyk2-ShC | TGA GGT TGG CTC AGC AGA ACG CTG TGA CA |
| Src-ShB | AGC GGC TCT GTA TGG CAG GTT CAC CAT CA |
| Src-ShC | GGA GGC TTC AAC TCC TCG GAC ACT GTC AC |
| Scrambled | GCA CTA CCA GAG CTA ACT CAG ATA GTA CT |

**Supplementary File 3. shRNA sequences targeting rat Pyk2 and Src**
