# Supplementary material for: α1-Adrenergic receptor–PKC–Pyk2–Src signaling boosts L-type Ca2+ channel CaV1.2 activity and long-term potentiation in rodents
Source: eLife. 2023 Jun 20;12:e79648. doi: 10.7554/eLife.79648 (PMC10325713; doi:10.7554/eLife.79648)
Supplement: Supplementary file 4. [file elife-79648-supp4.docx]

**Supplementary File 4.**

| **Plasmid** | **Orientation** | **Base pairs** | **Primer sequence** |
| --- | --- | --- | --- |
| pVETL-eGFP | Forward | 1131-1149 | CTA AGG TTG GTT ATT TGC G |
| pGFP-C-shLenti | Forward | 3074-3095 | TTG AGA TGC ATG CTT TGC ATA C |

**Supplementary File 4. Sequencing primers for validation of knockdown constructs**
